# Elevated Non-Esterified Fatty Acid Concentrations during Bovine Oocyte Maturation Compromise Early Embryo Physiology

**DOI:** 10.1371/journal.pone.0023183

**Published:** 2011-08-17

**Authors:** Veerle Van Hoeck, Roger G. Sturmey, Pablo Bermejo-Alvarez, Dimitrios Rizos, Alfonso Gutierrez-Adan, Henry J. Leese, Peter E. J. Bols, Jo L. M. R. Leroy

**Affiliations:** 1 Laboratory for Veterinary Physiology and Biochemistry, Department of Veterinary Sciences, Faculty of Biomedical, Pharmaceutical and Veterinary Sciences, University of Antwerp, Wilrijk, Belgium; 2 Hull-York Medical School, University of Hull, Hull, United Kingdom; 3 Departamento de Reproducción Animal y Conservación de Recursos Zoogenéticos, INIA, Madrid, Spain; University of Kansas Medical Center, United States of America

## Abstract

Elevated concentrations of serum non-esterified fatty acids (NEFA), associated with maternal disorders such as obesity and type II diabetes, alter the ovarian follicular micro-environment and have been associated with subfertility arising from reduced oocyte developmental competence. We have asked whether elevated NEFA concentrations during oocyte maturation affect the development and physiology of zygotes formed from such oocytes, using the cow as a model. The zygotes were grown to blastocysts, which were evaluated for their quality in terms of cell number, apoptosis, expression of key genes, amino acid turnover and oxidative metabolism. Oocyte maturation under elevated NEFA concentrations resulted in blastocysts with significantly lower cell number, increased apoptotic cell ratio and altered mRNA abundance of *DNMT3A*, *IGF2R* and *SLC2A1*. In addition, the blastocysts displayed reduced oxygen, pyruvate and glucose consumption, up-regulated lactate consumption and higher amino acid metabolism. These data indicate that exposure of maturing oocytes to elevated NEFA concentrations has a negative impact on fertility not only through a reduction in oocyte developmental capacity but through compromised early embryo quality, viability and metabolism.

## Introduction

Up-regulated lipolysis, a feature of metabolic disorders such as obesity and type II diabetes, results in increased plasma non-esterified fatty acid (NEFA) concentrations [1]–[4]. It is well established that elevated NEFA concentrations are cytotoxic for several cell types such as Leydig cells [5], nerve growth factor differentiated cells [6] and hepatocytes [7]. Furthermore, exposure of pancreatic β-cells to elevated NEFA concentrations impairs insulin secretion and is considered as an important factor in the pathogenesis of diabetes [8].

Using a bovine model we have demonstrated that elevated serum NEFA concentrations are reflected in the follicular fluid of the pre-ovulatory ovarian follicle [4] and can directly affect the granulosa cell viability and steroidogenesis [3], [9] as well as the developmental capacity of the oocyte [3], [4], [10]. In the human, saturated NEFA, such as Palmitic Acid (PA) and Stearic Acid (SA), suppress granulosa cell survival in a time-and-dose-dependent manner [11], which might impair fertility.

Epidemiological studies have shown that lipolysis-linked maternal metabolic disorders, such as obesity and type II diabetes [1], [2], are potential risk factors for reproductive disorders [12], [13], which could, at least in part, be related to early pregnancy loss [14], [15]. As such, obesity in women not only increases the risk of miscarriage but impairs the outcome of assisted reproductive technologies [16], [17], [18].

It is widely acknowledged that appropriate oocyte development governs several sequential and critical steps in meiosis, fertilization and early cleavage [19]–[21]. Furthermore, molecular events during oogenesis impact on the subsequent activation of the embryonic genome, manifested later in embryogenesis [22]–[24]. However, the extent of the relationship between these maternal metabolic disorders and subfertility is unclear.

In the present study, we hypothesized that elevated NEFA concentrations are a key metabolic factor in the relationship between maternal metabolic disorders and subfertility, through a negative effect on the oocyte. To address this, we have used a bovine oocyte in vitro culture model to investigate whether elevated NEFA concentrations during bovine oocyte maturation influence subsequent embryo phenotype.

We report that elevated NEFA concentrations, specifically Oleic Acid (OA), Palmitic Acid (PA) and Stearic Acid (SA), during oocyte maturation have negative consequences for the resulting preimplantation embryo, measured 8 days later at the blastocyst stage. These include altered blastocyst gene expression and reduced embryo quality, determined in terms of energy and amino acid metabolism; known markers of embryo viability. Our data provide evidence of a mechanism for metabolic deregulation appearing in the preimplantation embryo as a consequence of elevated NEFA concentrations during oocyte maturation and help to explain the higher rate of early pregnancy loss and miscarriage [15], [25] observed in women suffering metabolic disorders.

## Results

### Developmental Competence of Oocytes Matured in the Presence of elevated NEFA

Supplementing maturation medium with different combinations of NEFA had no significant effect on cleavage rates but based on the odds ratios, maturing oocytes in medium with elevated Stearic Acid (HIGH SA) or in medium supplemented with a combination of elevated Stearic Acid (SA), Palmitic Acid (PA) and Oleic Acid (OA) concentrations (HIGH COMBI) resulted in a significant reduction in the number of oocytes reaching the blastocyst stage at day 7 post insemination (p.i.) (*P*<0.05). The HIGH COMBI treatment also reduced the number of cleaved zygotes reaching the blastocyst stage compared with the control group (*P*<0.01). The capacity of cleaved zygotes to become blastocysts tended to be lower in the HIGH SA group (*P* = 0.06) compared with the control group. The data of the effects of HIGH SA and HIGH COMBI exposure during maturation on developmental competence are presented in Table 1.

**Table 1:** NEFA exposure of bovine oocytes significantly reduced the oocyte developmental competence and the quality of the resultant blastocysts.

n (%)	CONTROL	HIGH SA	HIGH PA	HIGH OA
**Oocytes**	**240**	**255**	**243**	**286**
**Cleaved**	133 (55.4)	126 (52.1)	155 (63.8)	194 (67.8)
**Blastocysts from** **Oocytes matured**	60 (25.0)^a^	45 (18.6)^b^	42 (17.3)^ b^	64 (22.4)^ a^
**Blastocysts from cleaved zygotes**	60 (45.1)^a^	45 (35.7)^a^	42 (27.1)^ b^	64 (33.0)^b^
**Blastocyst's cell** **number ± SD**	125.8±29.4^ a^	105.4±24.7^b^	118.5±34.5^ a^	122.7±23.9^ a^
**Blastocyst's apoptotic cell ratio ± SD**	0.09±0.05^a^	0.18±0.08^b^	0.20±0.12^b^	0.16±0.08^ b^

Oocytes (n = 1024; three replicates) were matured in maturation medium supplemented with 1) physiological NEFA  =  control (150 µM of total NEFA, i.e. OA, SA and PA); 2) elevated Stearic Acid  =  HIGH SA (75 µM SA); 3) elevated Palmitic Acid  =  HIGH PA (150 µM PA) and 4) elevated Oleic Acid  =  HIGH OA (200 µM OA). Data marked with different superscripts per row are significantly different between treatments (*P*<0.05).

### Cell Number and Apoptotic Cell Index in Bovine Blastocysts Arising from Oocytes Matured in the Presence of elevated NEFA

Total blastocyst cell number was significantly lower in the HIGH COMBI (104.7±26.1) and the HIGH SA (105.4±24.7) group compared with their control counterparts (125.8±29.4) (*P*<0.05). Furthermore, the apoptotic cell index was significantly higher in the HIGH SA embryos (0.18±0.08) compared with the control (0.09±0.05) and the HIGH COMBI embryos (0.14±0.12) (*P*<0.05). Data are listed in Table 1.

### Expression Patterns of Key Genes in Bovine Blastocysts Arising from Oocytes Matured in the Presence of elevated NEFA

**Figure 1:**
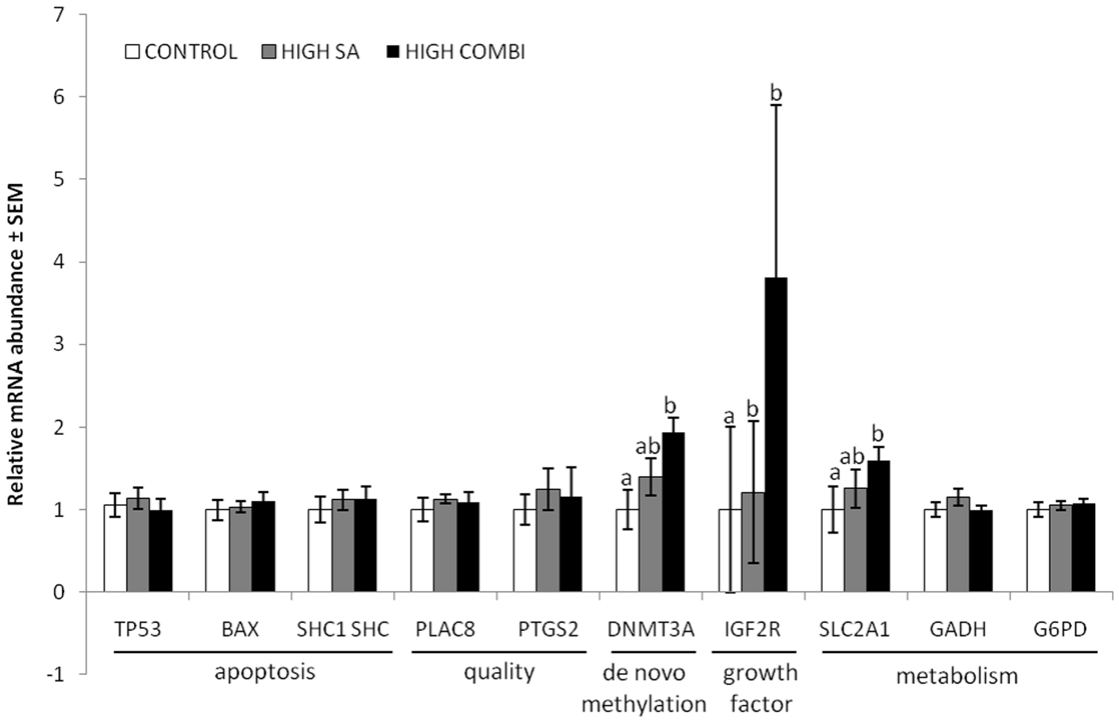
Expression patterns of key genes are altered in bovine blastocysts arising from oocytes matured in the presence of elevated NEFA. Blastocysts were derived from oocytes matured under control, HIGH SA and HIGH COMBI conditions (n = 192; five replicates). Bars with different superscripts are significantly different between treatments with P<0.05.

We next investigated the expression of key genes representative of cellular processes linked to apoptosis, development, quality and metabolism in the blastocyst. Surprisingly, the expression pattern of many of these genes did not differ between embryos arising from oocytes maturated with HIGH SA or the controls (Figure 1). However, these data did indicate differences in regulation of DNA methylation and glucose transport; both *DNMT3A* and *SLC2A1* were up-regulated in blastocysts originating from oocytes matured under HIGH COMBI conditions compared with the control group (*P*<0.05). The expression of the *IGF2R* gene was up-regulated in both groups matured under either HIGH SA or HIGH COMBI conditions, compared to the control group (*P*<0.05, Figure 1).

### Amino Acid Metabolism of Blastocysts Originating from NEFA-exposed Oocytes

**Figure 2:**
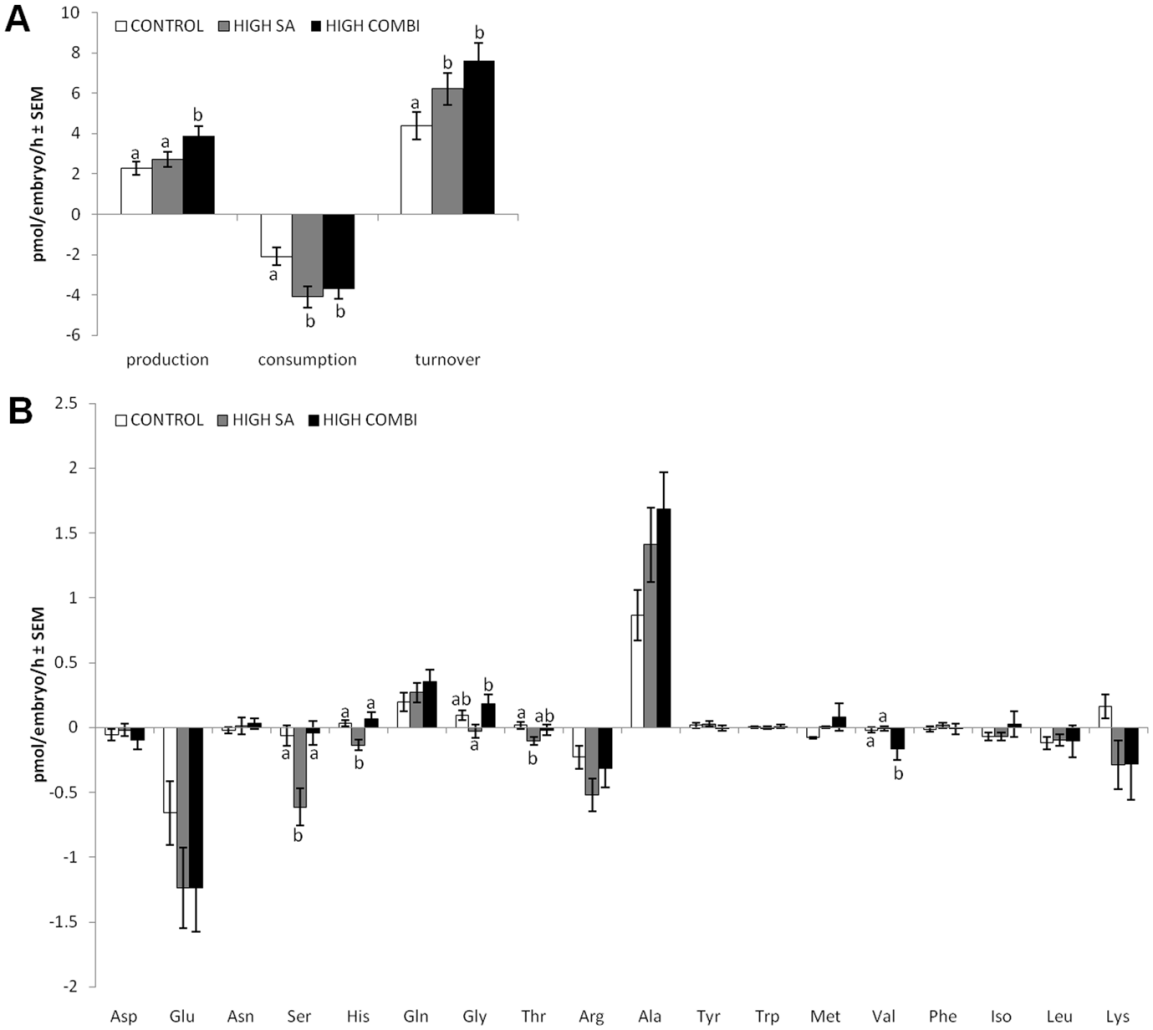
Amino acid metabolism of blastocysts originating from NEFA-exposed oocytes is compromised. A. Amino acid ‘turnover’ of day 7 blastocysts as calculated by summing all amino acids produced and consumed on a per-embryo basis. Comparison was done between blastocysts (n = 135; three replicates) derived from oocytes matured under control, HIGH SA and HIGH COMBI conditions. B. Overall mean profiles of individual amino acids by blastocyst in the three treatments. Bars with different superscripts are significantly different between treatments (P<0.05).

As shown in Figure 2A, blastocysts arising from oocytes matured in the presence of all three NEFA had a significantly elevated amino acid consumption (*P*<0.01), production (*P*<0.05) and overall turnover (*P*<0.01) compared with control embryos. HIGH SA embryos displayed significant higher amino acid consumption (*P*<0.01) and turnover (*P*<0.05) compared with control embryos (Figure 2A).

The profiles of individual amino acids showed that significantly higher levels of serine (*P*<0.01), histidine (*P*<0.01) and threonine (*P*<0.05) were depleted from the culture medium containing blastocysts derived from HIGH SA oocytes compared with control and HIGH COMBI embryos, whereas more valine (*P*<0.05) was depleted by HIGH COMBI blastocysts compared with control embryos (Figure 2B). Glycine concentrations decreased in the medium containing blastocysts from HIGH SA-exposed oocytes, suggesting consumption of glycine, whereas control and HIGH COMBI embryos showed net glycine production (*P*<0.05). Chi square tests revealed that there were no significant differences in developmental stage among treatment groups. Therefore, developmental stage can be excluded as possible confounder.

### Key Processes of ATP Production in Blastocysts Arising from Oocytes Exposed to NEFA

**Figure 3:**
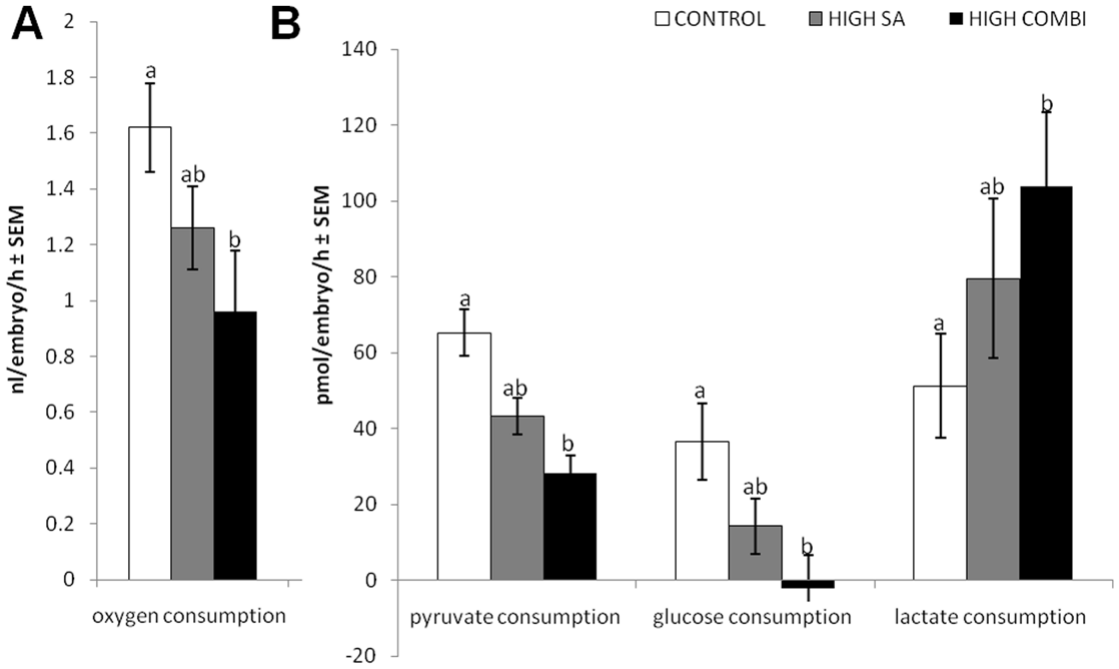
Key processes of ATP production are significantly altered in blastocysts arising from oocytes exposed to NEFA. A. Mean oxygen consumption of day 7 blastocysts. Blastocysts (n = 66; three replicates) were derived from control-, HIGH SA- and HIGH COMBI-exposed oocytes. Different superscripts indicate significant differences between treatments (P<0.05). B. Mean pyruvate, glucose and lactate consumption of blastocysts. Blastocysts (n = 84; five replicates) originated from oocytes matured in control, HIGH SA and HIGH COMBI maturation medium. Different superscripts indicate significant differences between treatments (P<0.05).

Given the observations that exposing oocytes to elevated NEFA impacted on blastocyst quality, gene expression patterns and amino acid metabolism, we next investigated the oxygen consumption by these embryos. Using a non-invasive real time assay of oxygen consumption, we found that blastocysts arising from oocytes matured under HIGH COMBI conditions consumed significantly less oxygen than control embryos (*P*<0.05, Figure 3A). There was no significant difference in oxygen consumption between the HIGH SA and the control group or between HIGH SA and HIGH COMBI embryos. We also determined the consumption of pyruvate, glucose and lactate by blastocysts originating from oocytes matured in the presence of NEFA. HIGH COMBI embryos consumed significantly less pyruvate and glucose when compared with control embryos (*P*<0.05, Figure 3B). By contrast, the HIGH COMBI embryos consumed significantly more lactate compared with the control group (*P*<0.05, Figure 3B). There was no significant difference in the level of pyruvate, glucose or lactate consumption between the HIGH SA and the control embryos. Chi square tests revealed that there were no significant differences in developmental stage among treatment groups. Developmental stage can therefore be excluded as a possible confounder.

## Discussion

Elevated serum NEFA concentrations, arising from up-regulated lipolysis, have been implicated as a key factor in the association between metabolic imbalances, cellular dysfunction and related pathologies [5]–[8]. In this context, we demonstrate, using a bovine model, that elevated NEFA concentrations during oocyte maturation have a profound negative impact on embryo quality; effects which persist some seven days after removal of NEFA exposure, in terms of blastocyst formation, viability, metabolism and gene transcription.

Support for the bovine as model of early mammalian reproduction, especially of the human, has grown steadily over the last decade. The human and bovine are single ovulators and there are close similarities between human and bovine ovarian function and oocyte characteristics, in contrast to major differences in ovarian physiology and reproductive function between rodents and humans [26]. Bovine and human embryos are remarkably similar with respect to microtubule timing of genome activation, metabolic requirements, interactions with the culture medium and duration of preimplantation development [27]–[29], which makes the cow an excellent model for human reproductive research.

Our data show that elevated NEFA exposure during oocyte maturation does not impair the ability of zygotes to reach the 2-cell stage. However, there is a reduction in the number of the HIGH NEFA-exposed oocytes capable of forming blastocysts. In bovine and human embryos, the developmental processes of the first 3-to-4 cleavage divisions occur under the control of maternally-derived mRNAs and proteins stored in the oocyte, until major embryonic genome activation (EGA) occurs at the 8–16 cell stage. The reduction in blastocysts formed indicates that NEFA-exposure during oocyte development has a significant negative impact on post-genome activated development as well as on the pattern of gene transcription after EGA. Data from recent studies on an obese female mouse model [30], [31] lend further support to the proposition that elevated NEFA concentrations contribute to the reduced fertility seen in females with compromised metabolic status.

The total cell number in blastocysts from HIGH NEFA-exposed oocytes was significantly reduced and the corresponding apoptotic cell ratio increased, compared with control counterparts. It is generally recognized that saturated NEFA, such as PA (C16:0) and SA (C18:0), can directly exert negative effects on cell viability [4], [5], [11]. In the present work, apoptotic cell ratio was significantly higher in blastocysts from oocytes matured in HIGH SA medium compared with those originating from oocytes matured in HIGH COMBI or control medium. One interpretation of these findings is that the unsaturated OA (C18:1), present in the HIGH COMBI mixture, may compensate for the negative effects induced by the saturated NEFA (PA and SA) in the COMBI mixture. This proposition is broadly in line with the recent findings of Aardema *et al*. [10], who recognized that OA prevents detrimental effects of saturated fatty acids.

NEFA exposure during oocyte maturation also affected the gene expression pattern of the resulting blastocysts. A significant increase in relative mRNA abundance of the HIGH COMBI embryos was found for the *SLC2A1* gene; a classic indication of cellular stress [32]. Furthermore, the *DNMT3A* gene was significantly up-regulated in HIGH COMBI blastocysts. This gene encodes the *de novo* DNA methyltransferase whose regulation is essential during preimplantation development for the proper establishment of epigenetic marks. Among the epigenetic events occurring during preimplantation development, the establishment of genomic imprinting is crucial for subsequent placentation and embryo development. *IGF2R* is a well known maternal imprinted gene whose dysregulation causes perturbed placentation and fetal growth [33]–[35]. In this regard, we observed that this gene was significantly up-regulated in HIGH COMBI embryos compared with the other treatments in the current study. No differences were found in the relative mRNA abundance of apoptosis related genes, such as *BAX*, *SHC1* and *TP53*, which may indicate that the increased apoptotic cell index observed in blastocysts may have originated earlier in development. Moreover, it has been proposed that translational and post-translational regulation of apoptosis related genes in embryos appears to be more important than transcriptional regulation [36].

In 2002, the ‘quiet embryo hypothesis’ was presented [37], which states that the most viable early embryos exhibit a ‘quieter’ metabolism, well-illustrated with regard to amino acids. This general pattern has been reported for bovine [38], porcine [39] and human embryos [40] and has been suggested as a general marker of embryo viability. Moreover, data on amino acid metabolism in cleavage stage embryos are predictive of the ability to form a blastocyst [38], with elevated metabolism indicating reduced embryo competence. We therefore determined the amino acid profiles of blastocysts arising from oocytes matured under the NEFA conditions. Our findings indicated that blastocysts from HIGH SA and HIGH COMBI had increased amino acid metabolism and were most likely to be less viable than the controls. The observation that in HIGH SA embryos the increased amino acid metabolism was accompanied by an increase in nuclear fragmentation, reflected in the higher apoptotic cell ratio, corroborates previous findings in bovine blastocysts that an elevated metabolism is associated with DNA damage [41].

It is increasingly recognized that oocyte and embryo metabolism are closely linked with subsequent developmental capacity [42]. The consumption of oxygen provides an indicator of global metabolic activity [43] and has previously been identified as an important viability indicator for bovine embryos [32]. In contrast with the up-regulated amino acid metabolism, we observed a reduced oxidative metabolism in the HIGH SA and HIGH COMBI embryos suggesting that oxygen consumption must be at a certain threshold to sustain appropriate levels of ATP, since chronically low ATP content can be responsible for implantation failure in seemingly normal human embryos [44], [45]. Our data in the bovine are broadly in line with the findings of Lopes *et al*. [46] that bovine blastocysts capable to giving rise to a pregnancy post-transfer have values for oxygen consumption in the mid range of the distribution and that a decrease in oxygen consumption is not conducive to embryo viability. Both the reduced oxygen consumption in HIGH SA and HIGH COMBI embryos and the concomitant reduction in pyruvate and glucose consumption in the same embryos (Figure 3) strongly suggest a drop in the rate of oxidative phosphorylation. Unusually, all of the blastocysts in the current work removed significant amounts of lactate from the medium, most probably due to the low amounts of glucose in our culture medium only present in the fetal calf serum. Lactate can serve as an alternative energy source and may sustain normal development to the blastocyst stage when glucose availability is low [47], [48].

Surprisingly, HIGH COMBI embryos consumed significantly more lactate compared with control embryos. One explanation for this might be that NEFA exposure results in an imbalance of the intracellular oxidation-reduction (REDOX) potential, as previously reported [49], [50]. Lactate has been recognized as a strong cytosolic reductant in mouse oocytes [51] and may thus be consumed by the HIGH COMBI embryos in our study to act as REDOX-regulator in the face of metabolic stress. Lactate can be converted by Lactate Dehydrogenase (LDH) into pyruvate, which may then enter the tricarboxylic acid cycle after conversion to oxaloacetate [52], [53], with the concomitant regeneration of NADH [47]. Alternatively, we observed elevated alanine production in HIGH COMBI embryos. It is plausible that the excess in lactate-derived pyruvate may be converted preferentially in the cytosol to alanine by alanine aminotransferase [54].

Such an alteration in REDOX status may affect the activity of REDOX sensitive transcription factors; for example, the amount of *SLC2A1* mRNA and the REDOX state are directly associated [48], [55]. Elevated Reactive Oxygen Species (ROS) concentrations are known to up-regulate *SLC2A1* transcription [32]; a pattern we have observed in the present study. Despite the increased expression of *SLC2A1*, also known as the facilitated glucose transporter 1, there was no difference in the glucose consumption by HIGH COMBI embryos; indeed there was no detectable glucose consumption by embryos in this group. *SCL2A1* up-regulation in the absence of glucose uptake could be caused by a failure in the *SLC2A1* protein production or impairment in the glucose transport mechanisms. Alternatively, the decreased glucose uptake in HIGH COMBI embryos may be considered in terms of the glucose:fatty acid cycle [56], that, in cardiac cells provision of NEFA, promotes fatty acid oxidation and inhibits glucose oxidation.

In conclusion, our data show that maternal metabolic conditions, associated with elevated NEFA during oocyte maturation, may compromise fertility through a reduction in oocyte developmental competence and the viability of the subsequent embryo.

In highlighting the metabolic problems associated with obesity and their potential relationship with subfertility, our findings are consistent with public health recommendations which emphasise the importance of women being at healthy weight before starting a pregnancy.

## Methods

For full details, see Materials and Methods S1, S2, S3, and Table S1.

### Composition of the Oocyte Maturation Treatments

The types and concentrations of free fatty acids used in the present study are based on bovine *in vivo* studies [4] and are physiologically appropriate, since circulating free fatty acid concentrations in women suffering lipolysis-linked metabolic disorders, including obesity [57], [58], are very similar to NEFA concentrations detected in bovine during an episode of up-regulated lipolysis [4].

Standard serum-free maturation systems are devoid of fatty acids, although the physiological environment, in which the oocyte matures *in vivo*, contains physiological, basal concentrations of NEFA [4]. In order to improve the relevance of our *in vitro* model, we therefore used a maturation medium supplemented with basal NEFA concentrations as control medium. We showed that inclusion of physiological concentrations of the key NEFA in maturation media formulations (three replicates; 595 oocytes) does not affect developmental competence compared to standard serum-free maturation media (P>0.1, details in Table 2).

**Table 2:** Validation of a physiological relevant control medium.

n (%)	standard serum-free maturation medium	physiologically relevant control medium
**Oocytes**	**291**	**304**
**Cleaved**	222 (76.2)^ a^	179 (64.8)^ a^
**Blastocysts from Oocytes matured**	88 (30.2)^ a^	89 (29.3)^ a^
**Blastocysts from Cleaved zygotes**	88 (39.6)^ a^	89 (49.7)^ a^

Cleavage rate at day 2 p.i., number of formed blastocysts at day 7 p.i. relative to the number of matured oocytes or to the number of cleaved zygotes. Oocytes (n = 595; three replicates) were matured in standard serum-free maturation medium devoid of all fatty acids [59] and in physiologically relevant, NEFA containing maturation medium with physiological, non toxic NEFA concentrations ( =  control medium used in this study: 150 µM of total NEFA, i.e. OA, SA and PA). Data marked with different superscripts per row are significantly different between treatments (*P*<0.05).

In preliminary experiments, we identified Stearic Acid (SA) as the most toxic NEFA for oocyte developmental competence (Table 1). The effect of elevated Palmitic Acid (PA) concentrations on developmental competence revealed similar findings as for elevated Stearic Acid (SA) treatment, though less pronounced (Table 1), supporting the findings of our previous study [4]. Elevated Oleic Acid (OA) treatment had no influence on developmental competence nor on total blastocyst cell number; only the blastocyst apoptotic cell index was significantly elevated after OA treatment during oocyte maturation.

For additional experiments we therefore focussed on the HIGH SA and the HIGH COMBI treatments. The following NEFA treatments were used in the present study:

1. **control**  =  physiological NEFA concentrations (150 µM total NEFA comprising 25 µM SA, 50 µM PA and 75 µM OA).
2. **HIGH SA**  =  elevated stearic acid concentrations (75 µM SA).
3. **HIGH COMBI**  =  combination of elevated NEFA concentrations (425 µM total NEFA, comprising 75 µM SA, 150 µM PA and 200 µM OA).

### Preparation of NEFA Treatments

All chemicals were purchased from Sigma®, unless otherwise stated. SA, PA and OA were dissolved in a stock solution of pure ethanol at concentrations of 25, 150 and 200 mM, respectively. These ethanol stock solutions were vortex-mixed for 4 min and diluted in working solutions to obtain the desired final concentration in maturation medium. The serum-free maturation medium consisted of TCM199 supplemented with 0.75% BSA free of fatty acids, 0.4 mM glutamine, 0.2 mM sodium pyruvate, 0.1 mM cysteamine, 50 µg/mL gentamycin and murine epidermal growth factor (mEGF, 20 ng/ml). All treatments were vigorously shaken for 45 min and filter-sterilised under aseptic conditions.

### *In Vitro* Embryo Production

*In vitro* production procedures were performed as previously described [59], using immature oocytes retrieved from cows collected within 2h of slaughter. For details refer to Materials and Methods S1, S2, S3, and Table S1.

### Oocyte Developmental Competence Assessment

Cleavage (2 days post insemination (p.i.)), and blastocyst rates (7 days p.i.) were defined as the number of cleaved zygotes or formed blastocysts per oocyte matured, respectively. The number of blastocysts from cleaved zygotes was also recorded.

### Measurement of Blastocyst Cell Number and Apoptotic Cell Index

Cell number and apoptotic cell index (number of apoptotic cells over total cell count) were assessed by staining normal and expanded day 7 blastocysts with propidium iodide (PI) and with terminal deoxynucleotidyl transferase dUTP nick end labeling (TUNEL), respectively, as described [60].

### Blastocyst RNA Extraction, Reverse Transcription and Quantification of mRNA Transcript Abundance

Molecular biology procedures were carried out as previously described [61]. See details in Materials and Methods S1, S2, S3 and Table S1.

### Amino Acid Profiling of Blastocysts

The amino acid content of spent culture media containing day 7 blastocysts was determined by reverse-phase HPLC as previously described [38]. Details in Materials and Methods S1, S2, S3 and Table S1.

### Oxygen Consumption Analysis of Blastocysts

Individual blastocysts day 7 p.i. were loaded into a PCR Glass micropipette (Drummond) and allowed to respire for 30 min to form an oxygen gradient. This oxygen gradient was measured in real time using a nanorespirometer (Unisense) and converted to oxygen consumption rate using SensorTrace Pro (Unisense) according to a previous study [62].

### Pyruvate, Lactate and Glucose consumption

Day 7 blastocysts were cultured individually for 3h in 4 µl droplets of modified SOF medium alongside empty control droplets. After the incubation period, the embryos were removed and spent culture droplets frozen at −80°C until analysis. Glucose, lactate and pyruvate utilization was determined by ultrafluorometric assays of spent medium as previously described [63].

### Statistical Analyses

All statistical procedures were carried out with SPSS 15.0 (for Windows, Chicago, IL, USA), unless otherwise stated. Cleavage and blastocyst rates were compared between the three treatments using a binary logistic regression model. For the other parameters a mixed model ANOVA, taking treatment as fixed factor and replicate as random factor, was used to compare differences between the three groups. No data transformations were necessary for inequality of variance between groups or for achieving normality for any data with the exception of amino acid metabolism. Chi square tests were used to analyze the effect of blastocyst developmental stage on oxygen, glucose, pyruvate and amino acid profiles. Relative transcript abundance was analyzed using the SigmaStat (Jandel Scientific) software package using one-way ANOVA with multiple pair-wise comparisons using Student-Newman-Kleus method post-hoc.

## Supporting Information

Materials and Methods S1 ***In Vitro*** **Embryo Production.**
(DOC)

Materials and Methods S2 **mRNA Expression Analysis.**
(DOC)

Materials and Methods S3 **Amino Acid Profiling.**
(DOC)

Table S1 **Details of primers used for qRT-PCR.**
(DOCX)
