# Supplementary material for: Elevated Non-Esterified Fatty Acid Concentrations during Bovine Oocyte Maturation Compromise Early Embryo Physiology
Source: PLoS One. 2011 Aug 17;6(8):e23183. doi: 10.1371/journal.pone.0023183 (PMC3157355; doi:10.1371/journal.pone.0023183)
Supplement: Materials and Methods S1 — In Vitro Embryo Production. (DOC) [file pone.0023183.s001.doc]

***In Vitro* Embryo Production**

Bovine ovaries were collected from culled dairy heifers at local slaughterhouses as soon as possible after slaughter, and transported immediately to the laboratory. They were then washed 3 times in warm saline solution (38°C) supplemented with 0.5% kanamycin. Subsequently, follicles with a diameter of 2-6 mm were aspirated. Only unexpanded cumulus oocyte complexes (COCs) surrounded by five or more cumulus cell layers (quality grade I) were matured *in vitro* as described before [59]. Briefly, the COCs were washed three times in Hepes-TALP, a salt solution with 114 mM NaCl, 3.1 mM KCl, 0.3 mM Na_2_HPO_4_, 2.1 mM CaCl_2_·2H_2_O, 0.4 mM MgCl_2_·6H_2_O, 2 mM bicarbonate, 1 mM pyruvate, 36 mM lactate, 1 M Hepes, 2 µL/mL phenol red, 0.4 mg/mL BSA and 50 µg/mL gentamycin. The COCs were first washed in 500 µL maturation medium and then matured in groups of 50-60 COCs in 500 µL maturation medium in 4-well plates (Nunc®, Langenselbold, Germany) for 22-24 h in humidified air with 5% CO_2_ at 38.5°C. After *in vitro* maturation (IVM), all COCs were coincubated per 100-120 with spermatozoa at a final concentration of 10^6^ sperm cells/ml for 20 h at 38.5 °C in fertilization medium in a humidified 5% CO_2_ incubator. For all experiments, frozen bull semen of proven fertility [59] was thawed and live spermatozoa were selected by centrifugation on a discontinuous Percoll® gradient (90 and 45%, Amersham Biosciences, Roosendaal, The Netherlands). The final sperm-egg ratio was adjusted to 5000:1. Fertilization medium consisted of 114 mM NaCl, 3.1 mM KCl, 0.3 mM Na_2_HPO_4_, 2.1 mM CaCl_2_·2H_2_O, 0.4 mM MgCl_2_·6H_2_O, 25 mM bicarbonate, 1 mM pyruvate, 36 mM lactate, 2 µL/mL phenol red, 6 mg/mL BSA, 50 µg/mL gentamycin and 10 µL/mL heparin. After coincubation with spermatozoa, the presumptive zygotes were vortexed for 4 min to remove excess sperm and cumulus cells. After 3 wash steps with HEPES-TALP and modified SOF medium, presumptive zygotes were cultured per 25 ± 4 in 50 μl modified SOF medium with mineral oil overlay (modular incubator: 38,5 °C, 5% CO_2_, 5% O_2_ and 90% N_2_) until day 7 p.i.. The SOF medium consisted of 108 mM NaCl, 7.2 mM KCl, 1.2 mM KH_2_PO_4_, 0.8 mM MgSO_4_.7H_2_O, 0.6% v/v sodium lactate, 25 mM NaHCO_3_, 0.0266 mM phenol red, 0.73 mM sodium pyruvate, 1.78 mM CaCl_2_.2H_2_O, 0.34 mM trisodium citrate, 2.755 mM myoinositol, 3% v/v BME 50x, 1% v/v MEM 100x, 0.4 mM glutamine, 5% serum and 50 µg/mL gentamycin.

Embryos originating from oocytes matured in control, HIGH SA and HIGH COMBI maturation medium were labelled as control, HIGH SA and HIGH COMBI embryos, respectively.
