# Supplementary material for: Elevated Non-Esterified Fatty Acid Concentrations during Bovine Oocyte Maturation Compromise Early Embryo Physiology
Source: PLoS One. 2011 Aug 17;6(8):e23183. doi: 10.1371/journal.pone.0023183 (PMC3157355; doi:10.1371/journal.pone.0023183)
Supplement: Materials and Methods S2 — mRNA Expression Analysis. (DOC) [file pone.0023183.s002.doc]

**mRNA Expression Analysis**

Poly(A) RNA was extracted from eight groups of 8 blastocysts per experimental group following the manufacturer’s instructions, using the Dynabeads mRNA Direct Extraction KIT (Dynal Biotech) with minor modifications. Immediately after extraction, the reverse transcription (RT) reaction was carried out following the manufacturer’s instructions (Bioline), using poly(T) primer, random primers and the MMLV reverse transcriptase enzyme, in a total volume of 40 μl, in order to prime the RT reaction and to produce cDNA. Tubes were heated to 70°C for 5 min to denature the secondary RNA structure and then the RT mix was completed with the addition of 100 units of reverse transcriptase. They were then incubated at 42°C for 60 min to allow the reverse transcription of RNA, followed by 70°C for 10 min to denature the RT enzyme. The quantification of all mRNA transcripts was carried out by real time quantitative reverse transcription-polymerase chain reaction (qRT-PCR). For qRT-PCR, 4 groups of cDNA per experimental group, were used with two repetitions for all genes of interest. Experiments were conducted to contrast relative levels of each transcript and histone H2AFZ in every sample. PCR was performed by adding a 2 μl aliquot of each sample to the PCR mix, containing the specific primers, to amplify histone H2AFZ (*H2AFZ*), tumor protein p53 (*TP53*), Bcl-2-associated X protein (*BAX*), (Src homology 2 domain containing) transforming protein 1 (*SHC1 SHC*, also known as *P66*), placenta-specific 8 (*PLAC8*), prostaglandin G/H synthase-2 (*PTGS2*, also known as *COX2*), DNA (cytosine-5-) methyltransferase 3 alpha (*DNMT3A*), insulin-like growth factor receptor 2 (*IGFR2*), solute carrier family 2 (facilitated glucose transporter) member 1 (*SLC2A1*, also known as *GLUT1*), glyceraldehyde-3-phosphate dehydrogenase (*GAPDH*) and glucose-6-phosphate dehydrogenase (*G6PD*). Primer sequences and the approximate sizes of the amplified fragments of all transcripts are shown in Table S1. For quantification, real time PCR was performed as previously described [61]. PCR conditions were optimized to achieve efficiencies close to 1 and then the comparative cycle threshold (CT) method was used to quantify expression levels. Quantification was normalized to the endogenous control. H2A.z. fluorescence was acquired in each cycle to determine the threshold cycle or the cycle during the log-linear phase of the reaction at which fluorescence increased above background for each sample. Within this region of the amplification curve, a difference of one cycle is equivalent to doubling of the amplified PCR product. According to the comparative CT method, the ΔCT value was determined by subtracting the *H2AFZ* CT value for each sample from each gene CT value of the sample. Calculation of ΔΔCT involved using the highest sample ΔCT value (i.e., the sample with the lowest target expression) as an arbitrary constant to subtract from all other ΔCT sample values. Changes in the relative gene expression of the target were determined using the formula 2-ΔΔCT.
