# Supplementary material for: Elevated Non-Esterified Fatty Acid Concentrations during Bovine Oocyte Maturation Compromise Early Embryo Physiology
Source: PLoS One. 2011 Aug 17;6(8):e23183. doi: 10.1371/journal.pone.0023183 (PMC3157355; doi:10.1371/journal.pone.0023183)
Supplement: Materials and Methods S3 — Amino Acid Profiling. (DOC) [file pone.0023183.s003.doc]

**Amino Acid Profiling**

Day 7 blastocysts were placed in 4 µl fresh, pre-equilibrated modified SOF medium, overlaid with mineral oil and cultured for 24 h, alongside empty medium control droplets; both the time of incubation and the stage of the embryo development were recorded. The embryos were then removed and the medium of the spent culture drops was frozen at -80°C until analysis for amino acid content within 21 days. A net fall in amino acid concentration in the spent culture droplet is interpreted as ‘consumption’; a net increase in concentration of an amino acid was interpreted as ‘production’. The sum of consumption and production is used as an overall indicator of amino acid metabolic activity and is defined as ‘turnover’.
