# Supplementary material for: Elevated Non-Esterified Fatty Acid Concentrations during Bovine Oocyte Maturation Compromise Early Embryo Physiology
Source: PLoS One. 2011 Aug 17;6(8):e23183. doi: 10.1371/journal.pone.0023183 (PMC3157355; doi:10.1371/journal.pone.0023183)
Supplement: Table S1 — Details of primers used for qRT-PCR. (DOCX) [file pone.0023183.s004.docx]

| **Gene** | **Primer sequence (5´-3´)** | **Fragment Size, bp** | **Gene Bank Accession No.** |
| --- | --- | --- | --- |
| ***H2AFZ*** | AGGACGACTAGCCATGGACGTGTG  CCACCACCAGCAATTGTAGCCTTG | 209 | NM_174809.2 |
| ***TP53*** | CTCAGTCCTCTGCCATACTA  GGATCCAGGATAAGGTGAGC | 364 | NM_174201.2 |
| ***BAX*** | CTACTTTGCCAGCAAACTGG  TCCCAAAGTAGGAGAGGA | 158 | NM_173894.1 |
| ***SHC1 SHC*** | GGTTCGGACAAAGGATCACC  GTGAGGTCTGGGGAGAAGC | 335 | NM_001075305.1 |
| ***PLAC8*** | CGGTGTTCCAGAGGTTTTTCC  AAGATGCCAGTCTGCCAGTCA | 166 | NM_001025325.1 |
| ***PTGS2*** | ATCTACCCGCCTCATGTTCCT  GGATTAGCCTGCTTGTCTGGA | 187 | NM_174445.2 |
| ***DNMT3A*** | CTGGTGCTGAAGGACTTGGGC  CAGAAGAAGGGGCGGTCATC | 317 | XM_867643.3 |
| ***IGF2R*** | GCTGCAGTGTGCCAAGTGAAAAAG  AGCCCCTCTGCCATTGTTACCT | 201 | NM_174352.2 |
| ***SCL2A1*** | CTGATCCTGGGTCGCTTCAT  ACGTACATGGGCACAAAACCA | 68 | NM_174602.2 |
| ***GAPDH*** | ACCCAGAAGACTGTGGATGG  AYGCCTGCTTCACCACCTTC | 247 | NM_001034034.1 |
| ***G6PD*** | CGCTGGGACGGGGTGCCCTTCATC  CGCCAGGCCTCCCGCAGTTCATCA | 347 | XM_583628.5 |
